# Sea Buckthorn Polysaccharide Ameliorates Colitis

**DOI:** 10.3390/nu16091280

**Published:** 2024-04-25

**Authors:** Qinqin Ouyang, Xin Li, Yongheng Liang, Rong Liu

**Affiliations:** 1 College of Life Sciences, Nanjing Agricultural University, Nanjing 210000, China; oyqq96@foxmail.com (Q.O.);; 2 College of Food Science and Technology, Nanjing Agricultural University, Nanjing 210000, China; 3 Department of Nutrition and Health, China Agricultural University, Beijing 100083, China

**Keywords:** gut microbiota, inflammation, inflammatory bowel disease, SCFAs, sea buckthorn polysaccharide

## Abstract

Ulcerative colitis (UC) is characterized by chronic inflammation and ulceration of the intestinal inner lining, resulting in various symptoms. Sea buckthorn berries contain a bioactive compound known as sea buckthorn polysaccharide (SBP). However, the precise mechanisms underlying the impact of SBP on UC remain unclear. In this study, we investigated the effects of pretreatment with SBP on colitis induced by DSS. Our findings demonstrate that SBP pretreatment effectively reduces inflammation, oxidative stress, and intestinal barrier damage associated with colitis. To further elucidate the role of SBP-modulated gut microbiota in UC, we performed fecal microbiota transplantation (FMT) on DSS-treated mice. The microbiota from SBP-treated mice exhibits notable anti-inflammatory and antioxidant effects, improves colonic barrier integrity, and increases the abundance of beneficial bacteria, as well as enhancing SCFA production. Collectively, these results strongly indicate that SBP-mediated amelioration of colitis is attributed to its impact on the gut microbiota, particularly through the promotion of SCFA-producing bacteria and subsequent elevation of SCFA levels. This study provides compelling evidence supporting the efficacy of pre-emptive SBP supplementation in alleviating colitis symptoms by modulating the gut microbiota, thereby offering novel insights into the potential of SBP as a regulator of the gut microbiota for colitis relief.

## 1. Introduction

Inflammatory bowel disease (IBD) encompasses chronic inflammatory disorders affecting the digestive system, including ulcerative colitis (UC) and Crohn’s disease (CD) [1,2]. Distinguishing between UC and CD relies on their distinct histological features, with UC primarily affecting the colon and rectum, while CD involves the entire gastrointestinal system, from the oral cavity to the anal canal [3]. Over the past years, UC and CD have emerged as significant global challenges in the field of gastroenterology [4,5,6]. The development of IBD, including UC and CD, is thought to arise from a complex interplay of multiple factors, including genetic and environmental influences. Although specific genes have been linked to an increased susceptibility to IBD, the underlying mechanisms by which these genes contribute to disease development are still under investigation [2]. In addition to genetic factors, environmental elements such as diet, gut microbial composition, and exposure to certain pathogens or toxins are recognized as significant contributors to the onset and exacerbation of IBD. However, establishing a definitive causal relationship between these factors and IBD onset remains a challenge.

Recent studies have provided compelling evidence highlighting the crucial role of the gut microbiome in the initiation and progression of IBD [7]. Individuals with IBD exhibit a notable disruption in the gut microbiota, characterized by a significant reduction in short-chain fatty acids (SCFAs)-producing bacterial species [8,9]. Given the well-documented immunomodulatory properties of SCFAs [10,11], the absence of SCFAs-producing bacteria could contribute to chronic inflammation and tissue damage in individuals with IBD [12,13]. Consequently, there has been a growing interest in exploring the potential therapeutic benefits of modulating the intestinal microbiome and its byproducts in the context of IBD [14,15,16]. One particularly promising approach is fecal microbiota transplantation (FMT), which involves transferring fecal matter from a healthy donor to an IBD patient to establish a more diverse and balanced gut microbiota in the recipient [17,18]. Additionally, accumulating epidemiological evidence highlights the significant impact of dietary factors on the development of chronic diseases, including IBD [16,19,20,21]. Therefore, investigating the role of diet in relation to IBD risk and treatment is an essential area of ongoing research. Further investigations are necessary to fully elucidate the intricate interactions among the intestinal microbiome, dietary factors, and disease development, with the ultimate goal of developing effective and personalized approaches for managing and preventing IBD.

Originating from the Himalayan region, sea buckthorn has spread throughout Eurasia and has a long history of use as both medicine and food by Tibetans and Mongolians [22,23]. Sea buckthorn polysaccharide (SBP) is a prominent functional component of sea buckthorn berries, accounting for up to 100 g/kg of dry weight and primarily consisting of starch, cellulose, hemicelluloses, and pectin. SBP has been demonstrated its ability to improve liver health by regulating inflammation, exerting antioxidant properties, and providing various protective effects [24]. Moreover, supplementation with SBP has shown positive effects in ameliorating gut microbial dysbiosis and improving intestinal barrier impairments induced by a high-fat diet in mice [25]. Although several studies have explored the diverse biological activities of SBP, the precise role and mechanism of SBP in mitigating IBD remain uncertain.

Based on its antioxidant and anti-inflammatory properties, we hypothesize that SBP has the potential to alleviate colitis. Moreover, considering the possible connection between SBP and the gut microbiota, we propose that the primary influence of SBP lies in modulating the gut microbiota and its metabolites, leading to a reduction in inflammation and oxidative stress. To investigate the effects of SBP on colitis, we conducted two animal trials. Firstly, we evaluated the efficacy of pre-supplementation with SBP in relieving symptoms in a DSS-induced colitis model. Subsequently, to confirm the impact of SBP-modulated gut microbiota on colitis, we performed fecal microbiota transplantation (FMT) from mice supplemented with SBP to mice with colitis. Through this experimental approach, we aimed to uncover the potential mechanisms by which SBP exerts its therapeutic benefits on colitis.

## 2. Materials and Methods

### 2.1. Animals Treatments and Sample Collection

Male C57BL/6J mice (7 weeks old) obtained from Vital River Animal Breeding were housed at the Animal Experiment Center of Nanjing Agricultural University under standard laboratory conditions. The mice were kept in groups of five per cage, maintained on a 12-h light/dark cycle at a temperature of 25 ± 2 °C, and provided with a humidity level of 50 ± 5%.

**Figure 1:**
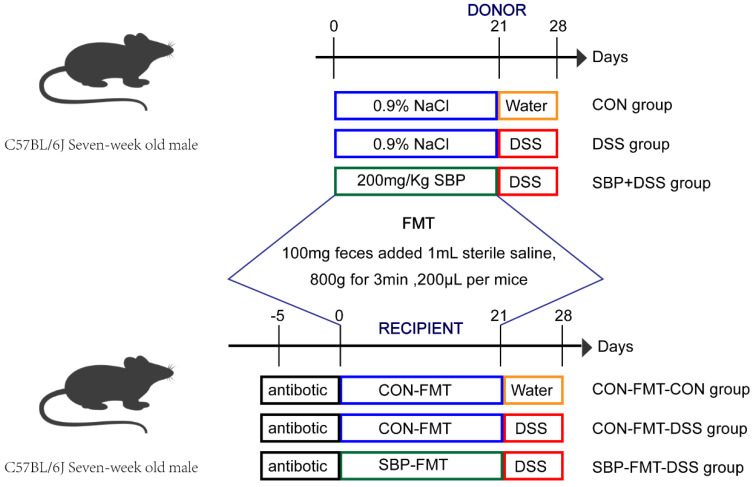
The study’s experimental design.

The experimental design employed in this study is presented in Figure 1. After a week of acclimation, the mice were divided into three groups, each consisting of ten mice. They were then orally administered either 200 μL of sterile saline or an equal amount of sterile saline containing 200 mg/kg body weight of SBP on a daily basis for three weeks. The dosage of sea buckthorn polysaccharide administered was established in reference to the optimum dose identified in prior research for ameliorating acute liver injury [24]. The treatment groups included the following: CON group and DSS group, which received oral administration of sterile saline for three weeks; SBP+DSS group, which received oral administration of SBP for 3 weeks. In the fourth week, the DSS group and SBP+DSS group were given drinking water containing 2.5% DSS (MP Biologicals, Santa Ana, CA, USA) to induce acute colitis. Daily measurements of body weight, feed quality, and disease activity index (DAI) were recorded from day 22 to day 28. The DAI, a combination of scores for weight loss (%), degree of diarrhea, and blood in stool, was used to assess the severity of colitis [16]. On day 29, mice were euthanized under anesthesia for further analysis. Colon length was measured for each mouse, and a 0.5-cm segment of the mid-colon was collected and fixed in 4% paraformaldehyde (PFA) solution or Carnoy’s fluid. Stool samples were collected aseptically and immediately flash-frozen in liquid nitrogen for subsequent experiments. Plasma was also obtained from the blood and stored at −80 °C for subsequent cytokine level assessments.

### 2.2. Fecal Microbiota Transplantation

To perform fecal transplants, a substantial amount of feces from mice treated with SBP or sterile saline was collected during the final three days of treatment, rapidly frozen, and stored at −80 °C. The collected stools, weighing 100 mg, were suspended in 1 mL sterile saline, stirred for 10 s, and then subjected to centrifugation at 800 rpm for 3 min. The supernatant was collected and used as the transplant material. The preparation of the transplant material was performed within a 30-min window prior to oral administration to minimize potential alterations in bacterial composition [26]. The transplantation procedure involved administering 200 μL of the transplant material orally every day for 3 weeks. In the fourth week, the CON-FMT-DSS group and SBP-FMT-DSS group were given drinking water containing 2.5% DSS.

To eliminate indigenous microorganisms, SPF mice were administered sterile saline containing vancomycin (50 mg/kg), neomycin (100 mg/kg), ampicillin (100 mg/mL), and metronidazole (100 mg/kg) twice a day for 5 consecutive days, as described previously [27].

### 2.3. Histological Analysis

Colon tissues were fixed with 4% PFA, sectioned, and subjected to hematoxylin and eosin staining (H&E) for visualizing tissue structures. Various parameters, including inflammatory infiltration, alterations in crypt structure, occurrence of ulcers and crypt loss, and degree of edema, were assessed. Histological scoring of these sections was based on edema level (0–1), degree of crypt damage (0–4), inflammatory infiltration (0–5), and ulceration (0–3) [16]. The fixed colon tissues in 4% PFA were also used for immunofluorescence staining and immunohistochemistry assays to detect and evaluate the expression levels of Muc2. Alcian blue staining was performed on fixed colonic tissues to quantify goblet cells. Images were obtained using a microscope [21].

### 2.4. Measurements of Antioxidant and Inflammation Indexes

Commercial kits (Jiancheng Bioengineering Institute, Nanjing, China) were used to quantify inflammatory mediators and representative redox enzymes, including MDA, T-AOC, T-SOD, CAT, and MPO, in colon tissues or plasma.

### 2.5. RNA Extraction and Quantification of Gene Expression

RNA extraction from collected colon tissue samples was carried out using TsingZol Reagent (tsingke), and the concentration was determined using a spectrophotometer (NanoDrop 2000, Thermo Fisher, Waltham, MA, USA). Reverse transcription of the extracted RNA into cDNA was performed using the SweScript All-in-One RT SuperMix (Sevier Biotechnology, Wuhan, China), along with gDNA removal. Quantitative RT-qPCR analysis was conducted using SYBR Green Master Mix (Sevier Biotechnology, Wuhan, China). The expression levels of target genes were normalized to the expression of GAPDH and calculated using the 2^−ΔΔCt^ method. The specific primers utilized are listed in Table 1.

### 2.6. Quantifcation of SCFAs Profiles

Targeted metabolomics was employed to quantify SCFAs in fecal samples. The analysis included the measurement of acetate, propionate, butyrate, caproate, isovalerate, and valerate. Fecal samples weighing 25 milligrams were dissolved in an appropriate solution, thoroughly mixed, and then centrifuged at 25,000 rpm for 2 min. The supernatant was diluted (1:50), passed through a sterile membrane, and stored in a 2 mL vial with a screw cap.

SCFAs in the fecal samples were analyzed using the Waters Iclass-AB Sciex 6500 (Agilent, Santa Clara, CA, USA) liquid mass spectrometry system. The Waters BEH C18 (Agilent, Santa Clara, CA, USA) chromatographic column was maintained at a temperature of 40 °C. The mobile phase consisted of two components: Phase A, composed of H_2_O + 0.1% FA, and Phase B, composed of ACN + 0.1% FA. For mass spectrometry analysis, the following parameters were set: ion source: electrospray ionization (ESI); detection mode: negative ion mode; ion source temperature: 450 °C; detector voltage: 4200 V.

### 2.7. Microbial Community Analysis

Bacterial DNA was extracted from frozen mouse stool samples using the E.Z.N.A soil DNA kit (Omega, GA, USA). PCR amplification was performed using genomic DNA samples (30 ng) and specific fusion primers to amplify the Vmin3-V4 hypervariable region. Following DNA amplification, purification was carried out using Agencourt AMPure XP (Beckman, Brea, CA, USA)magnetic beads to remove unwanted contaminants and ensure DNA fragment purity. Purified DNA products were eluted, labeled, and assessed for fragment range and concentration using the Agilent 2100 Bioanalyzer (Agilent, Santa Clara, CA, USA). High-throughput sequencing was performed on qualified libraries using the HiSeq platform (Illumina, San Diego, CA, USA). The sequencing reads underwent alignment and merging of overlapping regions to generate tags. These tags were then clustered into amplicon sequence variants (ASVs) and compared with databases for species annotation. ASVs and annotation results were used to analyze species complexity in the samples and compare species differences between groups. Various analyses, including diversity analysis, taxonomic profiling, and statistical comparisons, were conducted based on the ASVs and annotation data to gain insights into microbial composition and diversity in the samples.

### 2.8. Microbiota Data Analysis

The DADA2 (Divisive Amplicon Denoising Algorithm) method in the QIIME 2 software was used to denoise the original data and generate ASVs, which represent sequences that are 100% similar to each other. A feature table was generated, providing information on the abundance of each ASV in the dataset. Taxonomic assignment was performed using the Greengene database, enabling identification and classification of microbial taxa represented by the sequences in the dataset.

The R software (version 3.3.1) with the ‘VennDiagram’ package was used to create a Venn diagram for ASVs. PCoA analysis was conducted using QIIME (v1.80), with 75% of sequences randomly subsampled from the sample with the fewest sequences. The analysis was performed separately for weighted and unweighted taxonomic abundance and repeated 100 times for statistical robustness. Results were presented in a statistical analysis table and PCoA diagram. To identify biomarkers and reveal genomic features among different groups, LEfSe analysis (available at https://huttenhower.sph.harvard.edu/galaxy/, accessed on 22 June 2023) was employed, with only taxa meeting an LDA (linear discriminant analysis) threshold of 2.0 and average relative abundances exceeding 0.05% considered significant.

### 2.9. Statistical Analysis

All data were presented as means ± SEM and analyzed using GraphPad Prism 9.3 software. Statistical analysis was performed using one-way ANOVA or two-way ANOVA to compare data among groups. A *p*-value ≤ 0.05 was considered statistically significant.

## 3. Results

### 3.1. Prophylactic SBP Supplementation Attenuated Colitis Symptoms

**Figure 2:**
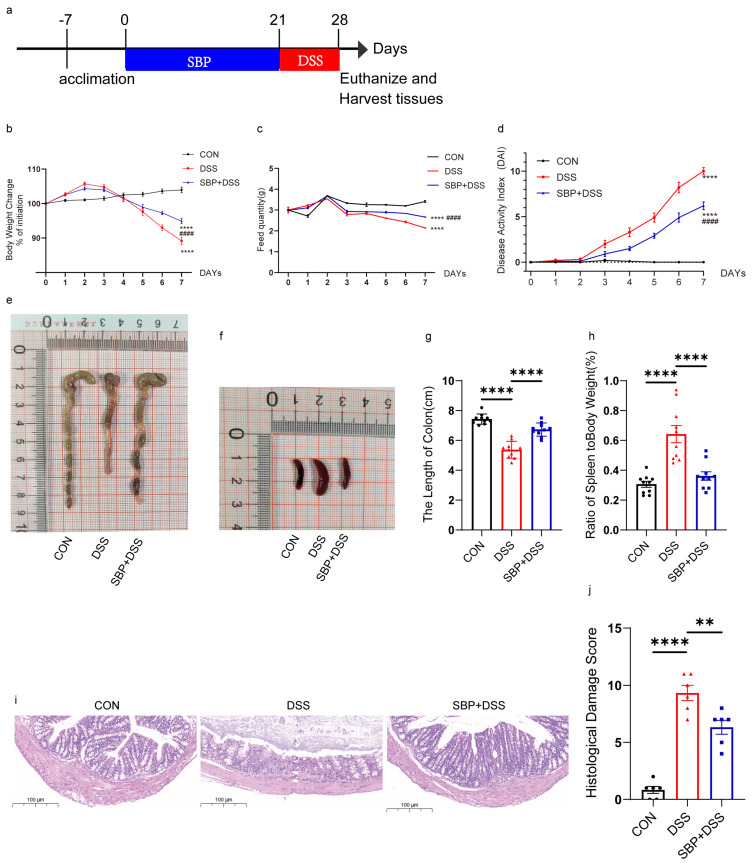
Prophylactic SBP effectively alleviated experimental colitis symptoms caused by DSS. (**a**) Diagram illustrating the experiment design. Oral sterile saline and SBP treatments are indicated. Body weight changes (**b**), daily food feed quality (**c**), and Disease Activity Index (DAI) scores (**d**) throughout the DSS−treated duration (n = 10). **** *p* ≤ 0.0001 relative to the CON group and ^####^ *p* ≤ 0.0001 relative to DSS group. Representative pictures of colons (**e**) and spleens (**f**). Colon length (**g**) and ratio of spleen/body weight (**h**) in each group (n = 10). **** *p* ≤ 0.0001. (**i**) Colon sections stained with H&E and (**j**) histological scores of colons (n = 6). ** *p* ≤ 0.01, **** *p* ≤ 0.0001.

To investigate the alleviating effect of SBP on DSS-induced colitis, we administered a daily oral dose of 200 mg/kg body weight of SBP to mice for 21 days. Subsequently, the mice were exposed to 2.5% DSS in drinking water for one week to induce acute colitis (Figure 2a). Throughout the study, we carefully monitored the mice on a daily basis to record disease symptoms, changes in body weight, and feed quality. After euthanizing the mice, we assessed the colonic pathology.

The administration of DSS resulted in several notable effects on the mice, including a reduction in body weight (Figure 2b), decreased feed consumption (Figure 2c), and shortened colon length (Figure 2e,g). Additionally, it led to an increase in the DAI score (Figure 2d) and the spleen/body weight ratio (Figure 2f,h). Prophylactic treatment with SBP significantly alleviated colitis symptoms in mice induced by DSS, as evidenced by several key observations. Firstly, SBP-treated mice experienced less weight loss compared to the group exposed to DSS alone, indicating the protective effect of SBP on weight maintenance during colitis (Figure 2b). Secondly, SBP administration increased the amount of feed consumed by the mice, suggesting an improved appetite and dietary intake (Figure 2c). Additionally, the DAI score, reflecting the severity of colitis symptoms, was significantly lower in the SBP-treated mice, indicating an improved overall condition (Figure 2d). Moreover, SBP-treated mice exhibited increased colon length, indicating reduced inflammation and tissue damage in the colon (Figure 2e,g). The spleen/body weight ratio was also reduced in mice receiving SBP treatment, reflecting decreased immune response and inflammation (Figure 2f,h). Representative histological sections stained with H&E (Figure 2i) and histopathology scores (Figure 2j) further demonstrated a notable reduction in colon severity with SBP intervention (*p* < 0.05). These results suggest that prophylactic SBP supplementation at a dosage of 200 mg/kg exerted vigorous impacts on the symptoms of DSS-induced colitis.

### 3.2. Prophylactic SBP Suppressed DSS-Induced Inflammation

**Figure 3:**
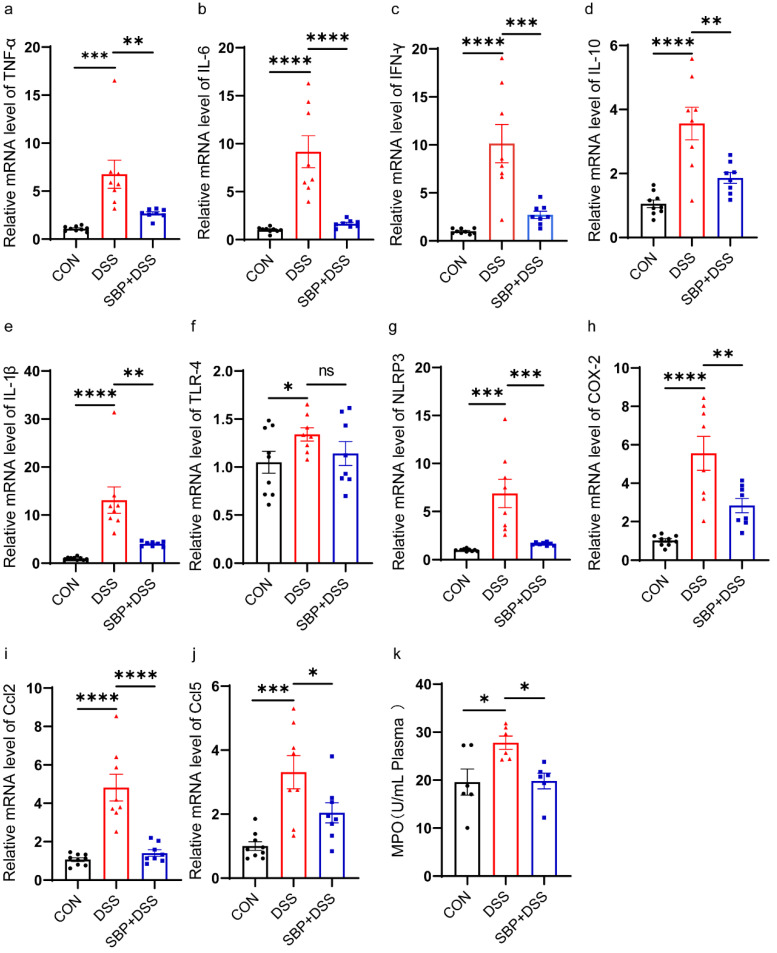
Prophylactic SBP ameliorates DSS-induced inflammation. The relative expression of inflammation-related genes in the colon. (**a**) TNF-α; (**b**) IL-6; (**c**) IFN-γ; (**d**) IL-10; (**e**) IL-1β; (**f**) TLR-4; (**g**) NLRP3; (**h**) COX-2; (**i**) Ccl2; (**j**) Ccl5(n = 8–10). (**k**) Concentrations of MPO in plasma (n = 8–10). **** *p* ≤ 0.0001, *** *p* ≤ 0.001, ** *p* ≤ 0.01, * *p* ≤ 0.05.

To evaluate the impact of SBP on inflammation in colitis, we measured the mRNA expression levels of various inflammation-related genes in colon tissue using RT-qPCR and the levels of myeloperoxidase (MPO) in plasma using a commercial kit. The results of the RT-qPCR analysis showed a significant increase in the relative expression levels of several inflammatory factors in the DSS group compared to the CON group. These factors included tumor necrosis factor-alpha (TNF-α, Figure 3a), interleukin 6 (IL-6, Figure 3b), interferon-gamma (IFN-γ, Figure 3c), interleukin 10 (IL-10, Figure 3d), interleukin 1-beta (IL-1β, Figure 3e), toll-like receptor 4 (TLR-4, Figure 3f), NLR family pyrin domain containing 3 (NLRP3, Figure 3g), cyclooxygenase-2 (COX-2, Figure 3h), chemokine C-C motif ligand 2 (CCL2, Figure 3i), and C-C motif ligand 5 (CCL5, Figure 3j). As expected, pretreatment with SBP obviously suppressed the relative expression of TNF-α, IL-6, IFN-γ, IL-10, IL-1β, NLRP3, COX-2, CCL2, and CCL5. TLR-4 expression also trended downward in the SBP+DSS group when compared to the DSS group, although this reduction did not reach statistical significance (Figure 3a–j). Additionally, the level of MPO was reduced in mice treated with prophylactic SBP compared to those treated with DSS alone (Figure 3k). These findings suggest that prophylactic SBP has the potential to ameliorate inflammation associated with ulcerative colitis.

### 3.3. Prophylactic SBP Ameliorated DSS-Induced Oxidative Stress

**Figure 4:**
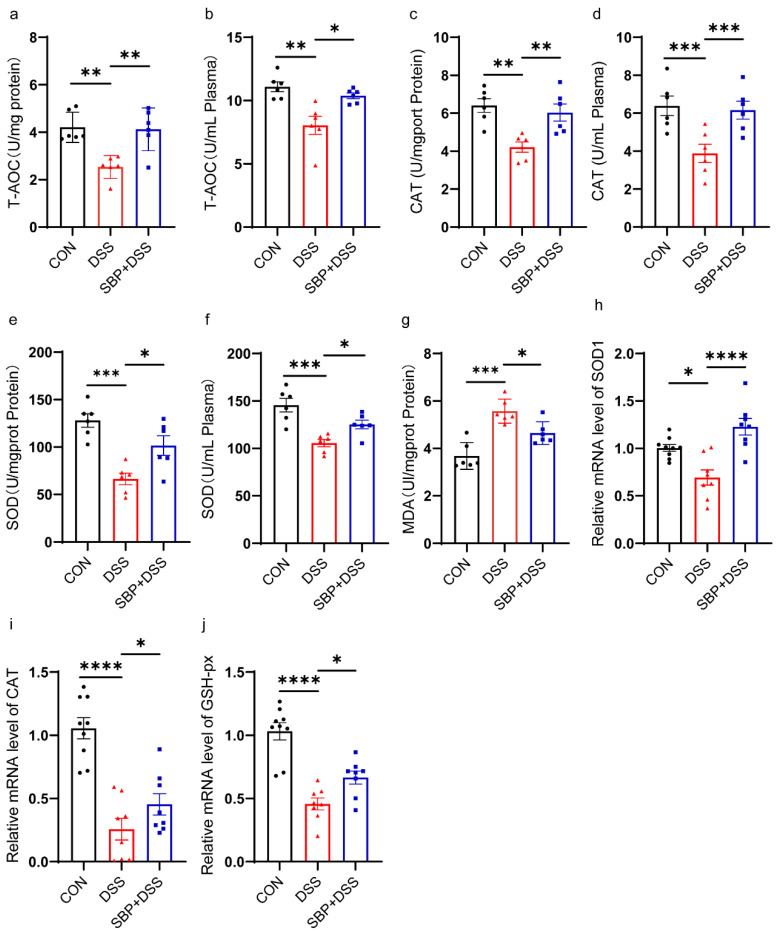
Prophylactic SBP attenuated oxidative stress. Capability of T-AOC in colon (**a**) and in plasma (**b**) (n = 6). Activity of CAT in colon (**c**) and in plasma (**d**) (n = 6). Activity of T-SOD in colon (**e**) and in plasma (**f**) (n = 6). (**g**) Concentrations of MDA in the colon (n = 6). The relative expression of antioxidation related gene in the colon. (**h**) SOD1; (**i**) CAT; (**j**) GSH-px (n = 8–10). **** *p* ≤ 0.0001, *** *p* ≤ 0.001, ** *p* ≤ 0.01, * *p* ≤ 0.05.

To evaluate the impact of SBP on oxidative stress in colitis, we measured the levels of total antioxidant capacity (T-AOC), catalase (CAT), and total superoxide dismutases (T-SOD) in both colon tissue and plasma, as well as the level of malondialdehyde (MDA) in the colon. DSS treatment resulted in a visible decrease in T-AOC levels in both the colon and plasma, which were mostly restored to normal states following SBP prophylaxis (Figure 4a,b). Figure 4c–f show that pretreatment with SBP significantly increased the activity of the antioxidant enzymes T-SOD and CAT compared to DSS treatment. Additionally, SBP prophylaxis normalized the elevated MDA levels in the colon caused by DSS treatment (Figure 4g). Furthermore, we evaluated the mRNA expression levels of antioxidant enzymes, including SOD1, CAT, and GSH-px, in the colon. We found an apparent reduction in their expression levels as a result of DSS treatment. However, with SBP prophylaxis, the expression of these antioxidant enzymes was largely restored to normal levels (Figure 4h–j). These data indicate that SBP effectively ameliorated oxidative stress in colitis.

### 3.4. Prophylactic SBP Improves Intestinal Integrity and Mucosal Barrier Function

**Figure 5:**
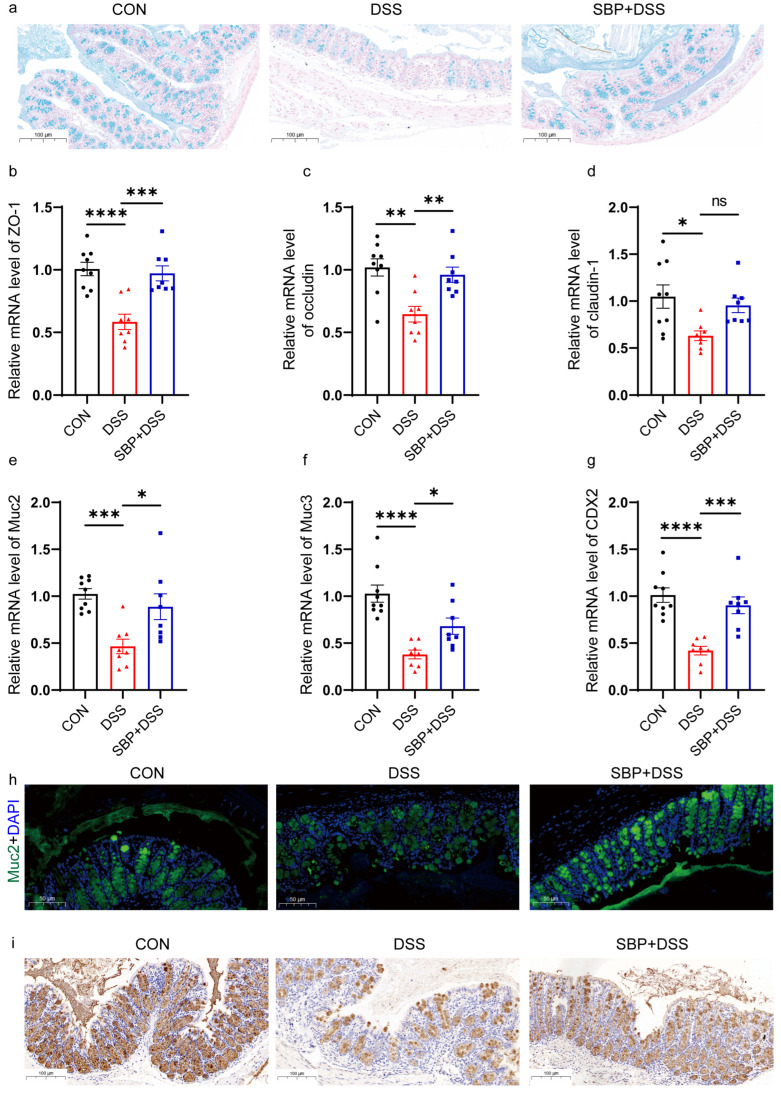
Prophylactic SBP recovered intestinal integrity damage. (**a**) Colonic sections stained with Alcian blue. The relative expression of intestinal integrity related genes in the colon. (**b**) ZO-1; (**c**) occludin; (**d**) claudin-1; (**e**) Muc2; (**f**) Muc3; (**g**) CDX2 (n = 8–10). **** *p* ≤ 0.0001, *** *p* ≤ 0.001, ** *p* ≤ 0.01, * *p* ≤ 0.05. (**h**) Muc2 immunofluorescent staining of colonic sections. (**i**) Muc2 immunohistochemistry staining of colonic sections.

Impairments in the intestinal barrier can result in heightened permeability, which is linked to the development of IBD [28]. In mouse models, colitis induced by DSS is characterized by reduced expression of tight junctions, subsequent increased intestinal permeability, and the onset of symptoms associated with inflammation of the colon [29,30]. To assess the impact of SBP supplementation on the colonic mucosal barrier, we quantified the levels of mucin in the colonic epithelia using Alcian blue staining. The results revealed that DSS administration resulted in a significant reduction in the thickness of the colonic epithelial mucosa (Figure 5a). However, pretreatment with SBP effectively mitigated this reduction, thereby restoring the mucosal barrier to normal levels (Figure 5a). We conducted RT-qPCR analysis to evaluate the expression levels of tight junction proteins, including ZO-1, occludin, and claudin-1, which are crucial for preserving the intestinal epithelial barrier. Additionally, we examined the expression of mucin proteins, specifically Muc2 and Muc3, responsible for producing the protective mucus layer in the intestine. We also assessed the relative expression of CDX2, an essential transcription factor protein of the intestinal epithelium. Supplementation with SBP significantly restored the levels of ZO-1 (Figure 5b), occludin (Figure 5c), Muc2 (Figure 5e), Muc3 (Figure 5f), and CDX2 (Figure 5g) in the colon of colitis mice, approaching near-normal levels. Although not statistically significant, there was a slight improvement observed in the expression level of claudin-1 (Figure 5d). To gain further insights into the effects of SBP on mucosal barrier functions, we assessed the expression of Muc2 using immunofluorescence staining and immunohistochemistry (Figure 5h,i). Consistent with the qPCR results, both immunofluorescence staining and immunohistochemistry demonstrated a significant decrease in Muc2 levels in DSS-treated mice, while pre-supplementation with SBP exhibited a protective effect, mitigating the decrease in Muc2 expression (Figure 5h,i). Overall, SBP pretreatment can counteract the detrimental effects of DSS-induced damage and promote the restoration of the colonic mucosal barrier.

### 3.5. Effects of SBP and DSS on Gut Microbiota

**Figure 6:**
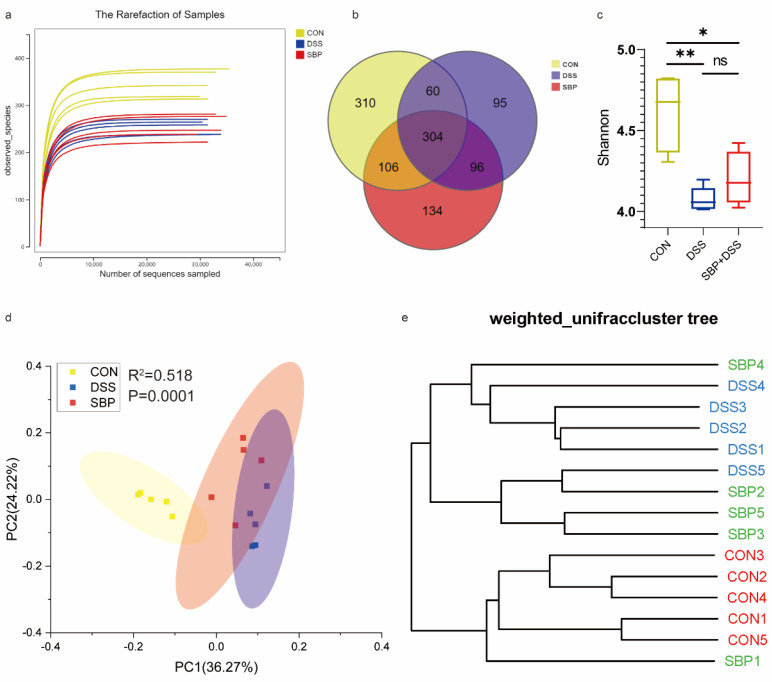
Prophylactic SBP modulated gut microbiota. (**a**) The alpha diversity rarefaction of samples. (**b**) Venn diagram of ASVs among groups. (**c**) Alpha diversity difference between groups shown by the Shannon index (n = 5). (**d**) Weighted UniFrac-based PCoA among groups. (**e**) The UPGMA cluster analysis among groups. * *p* ≤ 0.05, ** *p* ≤ 0.01.

Considering the crucial role of gut microbiota in maintaining intestinal homeostasis, we investigated the impact of SBP treatment on the composition and structure of the gut microbiota in mice with DSS-induced colitis. The rarefaction curves based on the ASVs level of the bacterial community displayed a flat trend, indicating reliable and comprehensive sequencing results due to sufficient sequencing depth employed in this study (Figure 6a). The Venn diagram shows a significant reduction in the number of ASVs in the DSS group compared to the CON group, whereas the SBP group exhibited an increase in ASVs compared to the DSS group (Figure 6b). Analysis of the Shannon indexes, indicators of alpha diversity, revealed that the DSS group had lower microbial diversity compared to the other two groups. Although SBP treatment increased the Shannon index, suggesting improved microbial diversity, there was no statistically significant difference in microbial diversity between the DSS group and the SBP+DSS group (Figure 6c). Weighted UniFrac-based PCoA demonstrated a distinct separation between the CON and DSS groups (*R*^2^ = 0.518, *p* = 0.0001, Figure 6d). Furthermore, prophylactic SBP treatment significantly influenced the composition of the gut microbiota, resulting in dissimilarity between the DSS and SBP+DSS groups (*R*^2^ = 0.278, *p* = 0.0464, Figure 6d). UPGMA cluster analysis further supported these findings, revealing a notable difference in the composition of the gut microbiota between the DSS and CON groups. However, SBP administration exerted a beneficial effect in mitigating this difference, leading to a composition of the gut microbiota more closely resembling the CON group (Figure 6e). These results indicate that SBP supplementation positively impacts the gut microbiota, potentially helping to restore it to a state more similar to that of healthy, non-colitis mice.

**Figure 7:**
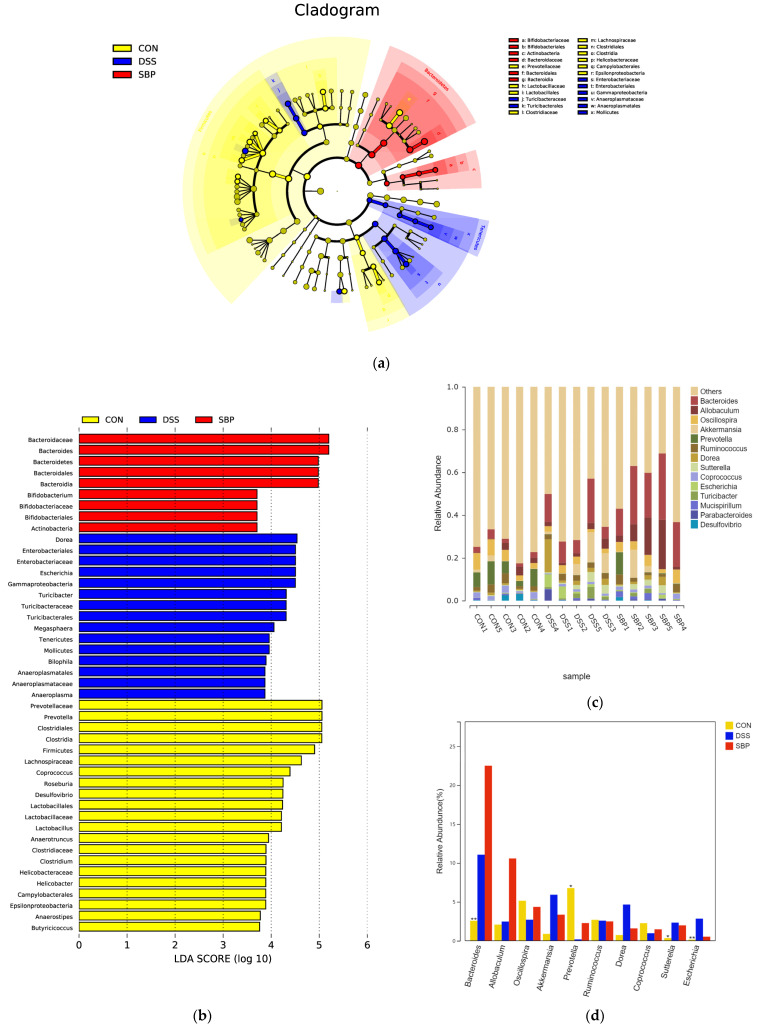
Prophylactic SBP altered gut microbial composition and structure. (**a**) LEfSe analysis illustrating the microbial features. (**b**) LDA score based on LEfSe analysis. (**c**) Main microbiota at the genus level for each sample. (**d**) Difference of microbiota at the genus level among groups. * *p* ≤ 0.05, ** *p* ≤ 0.01.

Subsequently, we conducted LEfSe-LDA analysis (Figure 7) to identify significant biomarkers or features distinguishing the three experimental groups. The results of the LEfSe analysis are presented in Figure 7a,b displays the LDA scores obtained from the analysis. Notably, prophylactic SBP administration resulted in an enrichment of *Bacteroides* and *Bifidobacterium* in the gut microbiota (Figure 7b). These bacterial genera are known for their ability to metabolize complex polysaccharides through fermentation, leading to the synthesis of short-chain fatty acids. Conversely, mice with colitis exhibited an enrichment of fifteen bacterial profiles, including *Escherichia* and *Turicibacter* (Figure 7b), which have been associated with gastrointestinal infections. The different patterns of enrichment observed in the gut microbiota emphasize the positive effects of SBP treatment in regulating the composition of the microbiota and fostering a more favorable gut environment. Figure 7c presents the primary microbial community at the genus level for each sample, while Figure 7d illustrates the alterations observed in the primary microbial community between groups. According to our research, the use of DSS led to a notable reduction in the prevalence of *Prevotella* and an increase in the populations of *Escherichia* and *Sutterella*. However, SBP treatment effectively nullified these alterations. Overall, the administration of SBP induced significant changes in the composition of the gut microbiota, accompanied by increased diversity. SBP treatment promoted the enrichment of beneficial bacteria and a reduction in the abundance of harmful bacteria; thus, suggesting its potential in restoring a healthier gut microbiota profile.

### 3.6. Effects of SBP and DSS on SCFAS

**Figure 8:**
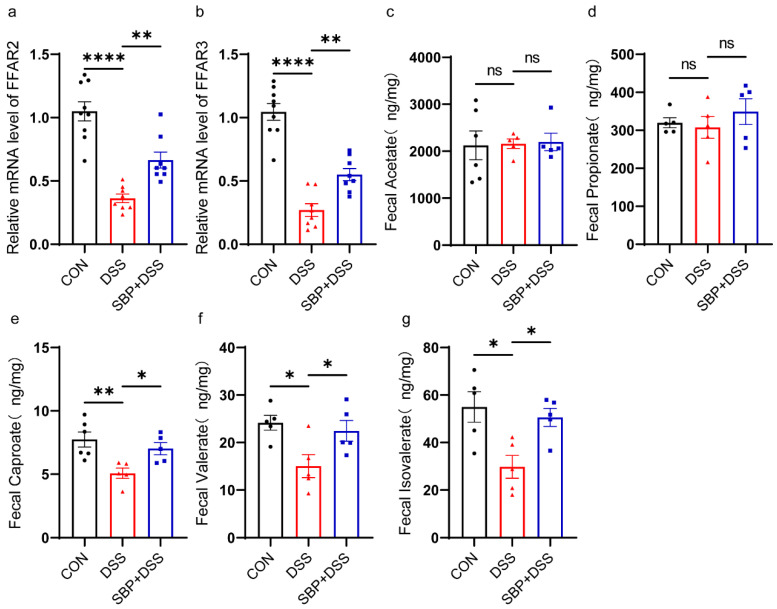
Prophylactic SBP improved the production of SCFAs. The relative expression of FFAR2 (**a**) and FFAR3 (**b**) in colon quantified by qPCR (n = 8–10). ** *p* ≤ 0.01, **** *p* ≤ 0.0001. Concentrations of fecal acetate (**c**), caproate (**d**), isovalerate (**e**), propionate (**f**), and valerate (**g**) (n = 5–6). * *p* ≤ 0.05, ** *p* ≤ 0.01.

To assess how SBP supplementation affects the metabolites produced by the gut microbiota, we evaluated the mRNA expression of FFAR2 and FFAR3, which are receptors primarily activated by SCFAs. The results indicated that pretreatment with SBP effectively restored the diminished levels of FFAR2 and FFAR3 in the colon observed in the DSS group (Figure 8a,b). Targeted metabolomics analysis was performed to quantify several SCFAs, including acetate, propionate, caproate, valerate, and isovalerate. These results indicated that the levels of acetate (Figure 8c) and propionate (Figure 8d) did not exhibit significant alterations, while caproate (Figure 8e), valerate (Figure 8f), and isovalerate (Figure 8g) were conspicuously lower in the DSS group. Prophylactic SBP administration partially rescued the reduction of these SCFAs. These findings suggest that prophylactic SBP administration could potentially regulate the metabolism of the gut microbiota, thereby attenuating DSS-induced colitis.

### 3.7. SBP-FMT Contributed to Alleviating Colitis Symptoms

**Figure 9:**
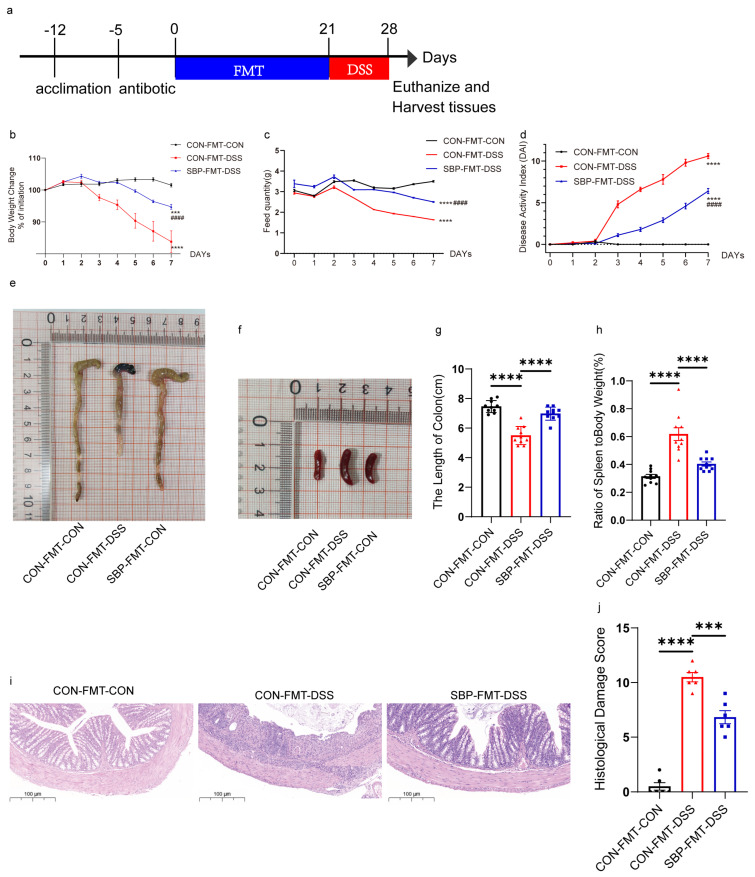
SBP-FMT effectively alleviated experimental colitis symptoms caused by DSS. (**a**) Diagram illustrating the experiment design. CON-FMT and SBP-FMT treatments are indicated. Body weight changes (**b**), daily food feed quality (**c**), and DAI scores (**d**) throughout the DSS-treated duration (n = 10). *** *p* ≤ 0.001, **** *p* ≤ 0.0001 relative to the CON-FMT-CON group and #### *p* ≤ 0.0001 relative to CON-FMT-DSS group. Representative pictures of colons (**e**) and spleens (**f**). Colon length (**g**) and ratio of spleen/body weight (**h**) in each group (n = 10). (**i**) Colon sections stained with H&E and (**j**) histological scores of colons (n = 6). *** *p* ≤ 0.001, **** *p* ≤ 0.0001.

To evaluate the effects of SBP-mediated microbiota on colitis, we performed a second animal trial by transplanting fecal microbiota from mice that underwent SBP gavage for 21 days, as shown in Figure 9a. Similar to the previous trial, the mice were monitored daily for disease symptoms, feed quality, and changes in body weight. Colonic pathology was evaluated after sacrificing the mice. Administration of DSS resulted in decreased body weight (Figure 9b), reduced food intake (Figure 9c), elevated DAI score (Figure 9d), shortened colon length (Figure 9e,g), and an elevated ratio of spleen/body weight in the mice (Figure 9f,h). SBP-FMT contributed to the improvement of colitis symptoms, as evidenced by a notable reduction in the DAI score (Figure 9d) and the ratio of spleen/body weight (Figure 9f,h). Additionally, SBP-FMT led to an increase in body weight (Figure 9b), feed quality (Figure 9c), and colon length (Figure 9e,g), indicating a positive impact on the overall health of mice with colitis. Representative H&E staining sections and histopathology scores are depicted in Figure 9i,j. The group receiving SBP-FMT exhibited significantly reduced pathological damage compared to the CON-FMT-DSS group. This was evidenced by the restoration of goblet cells to their normal state, substantial improvement in edema, and minimal infiltration of inflammatory cells. The histopathology scores also showed a significant improvement in the severity of inflammation due to SBP-FMT (*p* < 0.01). The results indicate that a 21-day treatment of SBP-FMT greatly improves the symptoms of colitis induced by DSS.

### 3.8. SBP-FMT Alleviated DSS-Induced Inflammation

**Figure 10:**
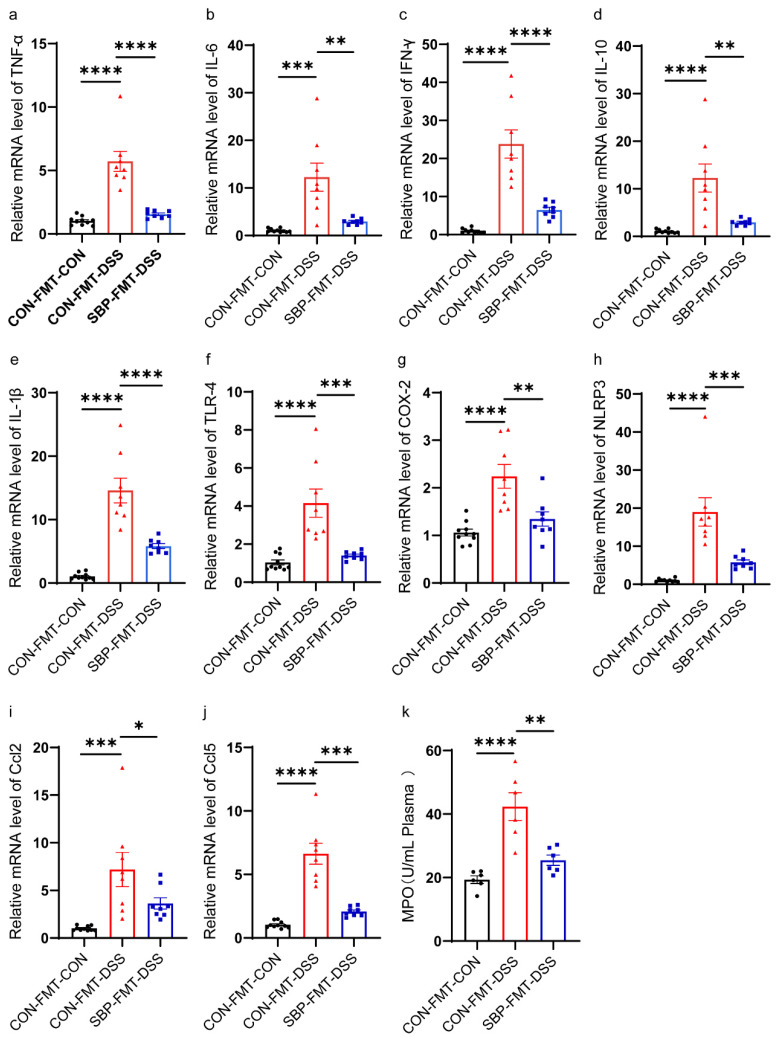
SBP-FMT ameliorates DSS-induced inflammation. The relative expression of inflammation related genes in the colon. (**a**) TNF-α; (**b**) IL-6; (**c**) IFN-γ; (**d**) IL-10; (**e**) IL-1β; (**f**) TLR-4; (**g**) NLRP3; (**h**) COX-2; (**i**) Ccl2; (**j**) Ccl5 (n = 8–10). (**k**) Concentrations of MPO in plasma (n = 8–10). **** *p* ≤ 0.0001, *** *p* ≤ 0.001, ** *p* ≤ 0.01, * *p* ≤ 0.05.

To evaluate the effects of SBP-mediated microbiota on systemic and intestinal inflammatory responses, we measured plasma MPO levels using a commercial kit and examined the mRNA expression of inflammation-related genes in the colon through RT-qPCR. The SBP-FMT-DSS group exhibited a significant reduction in the expression of TNF-α, IL-6, IFN-γ, IL-10, IL-1β, TLR-4, NLRP3, COX-2, CCL2, and CCL5 compared to the CON-FMT-DSS group (Figure 10a–j). Moreover, the SBP-FMT-DSS group showed a noticeable decrease in MPO levels in the plasma compared to the CON-FMT-DSS group (Figure 10k). These findings suggest that SBP-mediated microbiota can attenuate colitis-associated inflammation.

### 3.9. SBP-FMT Alleviated DSS-Induced Oxidative Stress

**Figure 11:**
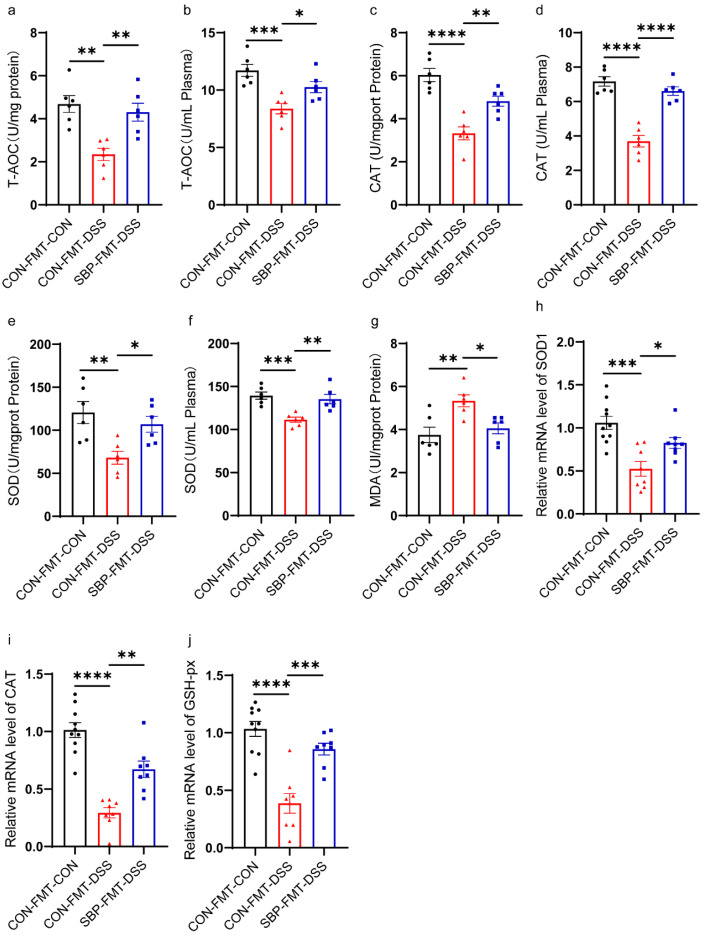
SBP-FMT attenuated oxidative stress. Capability of T-AOC in colon (**a**) and in plasma (**b**) (n = 6). Activity of CAT in colon (**c**) and in plasma (**d**) (n = 6). Activity of T-SOD in colon (**e**) and in plasma (**f**) (n = 6). (**g**) Concentrations of MDA in the colon (n = 6). The relative expression of antioxidation related genes in the colon. (**h**) SOD1; (**i**) CAT; (**j**) GSH-px (n = 8–10). **** *p* ≤ 0.0001, *** *p* ≤ 0.001, ** *p* ≤ 0.01, * *p* ≤ 0.05.

To evaluate the influence of SBP-mediated microbiota on oxidative stress, we examined the levels of T-AOC, the activity of T-SOD and CAT in both the colon and plasma, as well as the level of MDA in the colon. DSS significantly diminished T-AOC levels in both the colon and plasma, but SBP-FMT effectively restored and normalized the T-AOC levels (Figure 11a,b). Compared to the CON-FMT-DSS group, SBP-FMT notably decreased the activity of CAT and T-SOD in the colon and plasma (Figure 11c–f). Additionally, the levels of MDA in the colon, which were significantly elevated by DSS, returned to normal levels with SBP-mediated microbiota administration (Figure 11g). Furthermore, we evaluated the mRNA levels of SOD1, CAT, and GSH-px in the colon. DSS caused a notable decrease in their expression, but SBP-FMT treatment mostly reversed this to usual levels (Figure 11h–j). The findings suggest that microbiota regulated by SBP can alleviate oxidative stress in mice with colitis.

### 3.10. Regulation of SBP-FMT on Intestinal Integrity

**Figure 12:**
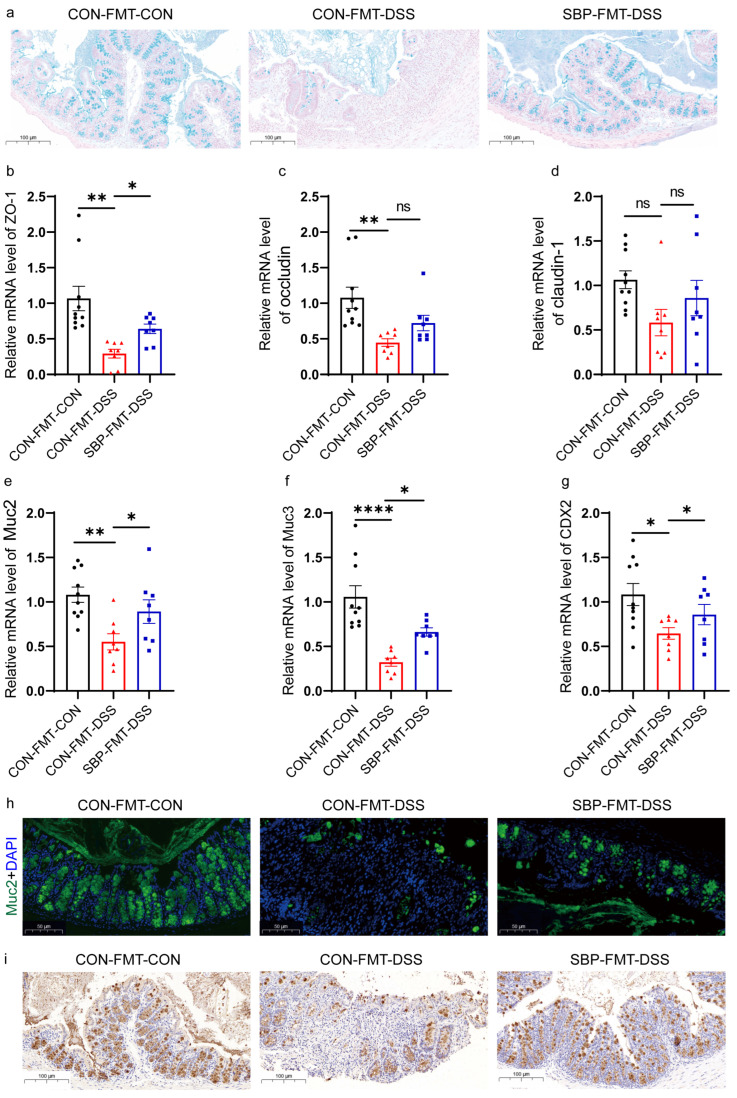
SBP-FMT recovered intestinal integrity damage. (**a**) Colonic sections stained with Alcian blue. The relative expression of intestinal integrity related genes in the colon. (**b**) ZO-1; (**c**) occludin; (**d**) claudin-1; (**e**) Muc2; (**f**) Muc3; (**g**) CDX2 (n = 8–10). **** *p* ≤ 0.0001, ** *p* ≤ 0.01, * *p* ≤ 0.05. (**h**) Muc2 immunofluorescent staining of colonic sections. (**i**) Muc2 immunohistochemistry staining of colonic sections.

The impact of SBP-mediated microbiota on the protective layer of the colon was assessed by examining the colonic epithelium of goblet cells that secrete mucin using Alcian blue staining. In contrast to the CON-FMT-DSS group, the SBP-FMT-DSS group exhibited a notable increase in the thickness of the colonic epithelial mucosa (Figure 12a). The RT-qPCR results indicated that SBP-FMT effectively elevated the expression of ZO-1 compared to the CON-FMT-DSS group (Figure 12b). Additionally, SBP-FMT treatment partially increased the levels of occludin and claudin-1 in the colon, although the differences were not statistically significant (Figure 12c,d). The levels of Muc2, Muc3, and their regulator CDX2 were also assessed using RT-qPCR. The qPCR results demonstrated that SBP-mediated microbiota can protect against the reduction of Muc2, Muc3, and CDX2 caused by DSS treatment (Figure 12e–g). Furthermore, we measured the expression of Muc2 in the colon through immunofluorescence staining and immunohistochemistry. Similar to the qPCR results, the immunofluorescence staining and immunohistochemistry also indicated that DSS treatment reduced the expression of Muc2 and SBP-FMT treatment had a protective effect (Figure 12h–i). In general, SBP-mediated microbiota can alleviate colonic mucosal barrier damage caused by DSS.

### 3.11. Impacts of SBP-FMT on the Gastrointestinal Microbiome

**Figure 13:**
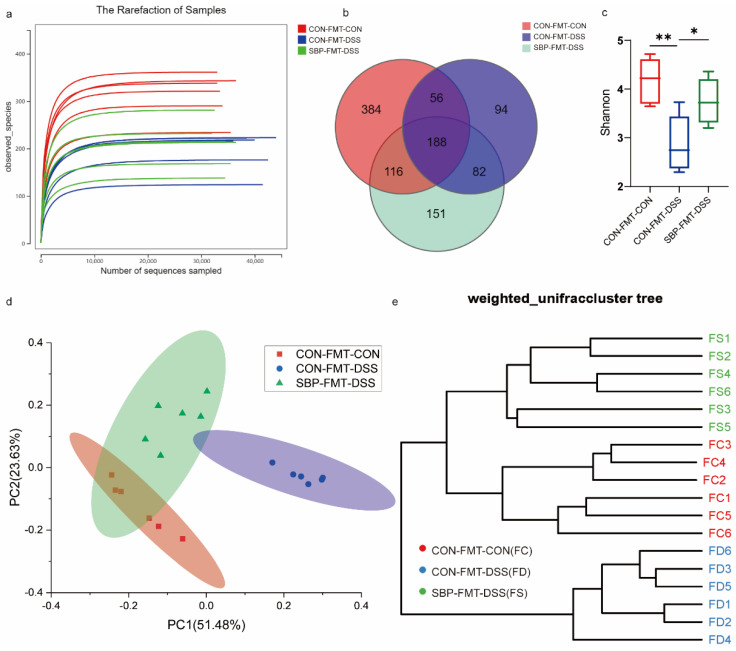
SBP-FMT modulated gut microbiota. (**a**) The alpha diversity rarefaction of samples. (**b**) Venn diagram of ASVs among groups. (**c**) Alpha diversity difference between groups shown by Shannon index (n = 6). * *p* ≤ 0.05, ** *p* ≤ 0.01. (**d**) Weighted UniFrac-based PCoA among groups. (**e**) The UPGMA cluster analysis among groups.

The gut microbiota composition after SBP-FMT treatment was assessed using 16S rRNA gene sequencing. As more reads were sampled, the rarefaction curves gradually reached a plateau, indicating that the sequencing depth was adequate for capturing the diversity of the gut microbiota (Figure 13a). In comparison to the CON-FMT-CON group, the CON-FMT-DSS group showed a noticeable reduction in the number of ASVs, whereas the SBP-FMT-DSS group displayed an increase in ASVs compared to the CON-FMT-DSS group (Figure 13b). Additionally, the CON-FMT-DSS group exhibited considerably reduced diversity according to the Shannon indexes in comparison to the other two groups (Figure 13c). The PCoA analysis illustrated a distinct distinction among the three groups, with the SBP-FMT-DSS group being in closer proximity to the CON-FMT-CON group (*R*^2^ = 0.684, *p* = 0.0001, Figure 13d). Similarly, the UPGMA cluster analysis revealed a conspicuous difference in the composition of the gut microbiota between the CON-FMT-DSS and CON-FMT-CON groups, which was mitigated by SBP-FMT treatment, resulting in a composition more similar to the CON-FMT-CON group (Figure 13e). The findings suggest that SBP-FMT treatment has a similar effect to prophylactic SBP treatment in modulating the composition and diversity of the gut microbiota.

**Figure 14:**
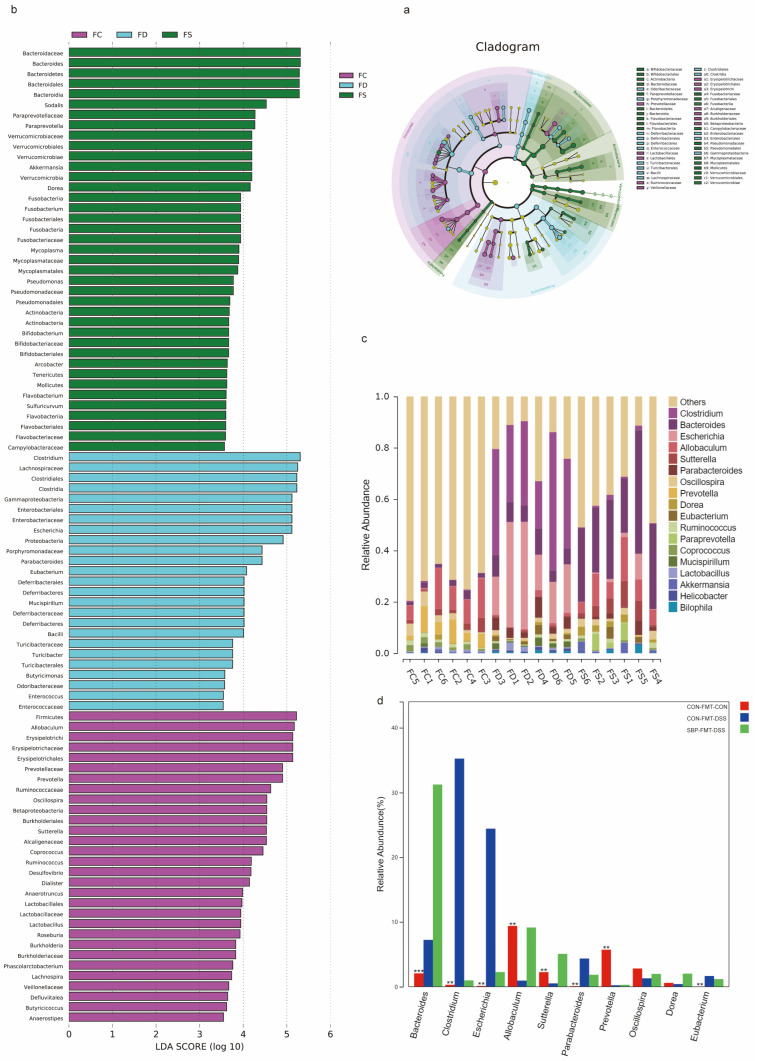
SBP-FMT altered gut microbial composition and structure. (**a**) LEfSe analysis illustrating the microbial features. (**b**) LDA score based on LEfSe analysis. (**c**) Main microbiota at the genus level of each sample. (**d**) Difference of microbiota at the genus level among groups. ** *p* ≤ 0.01, *** *p* ≤ 0.001.

Subsequently, we conducted LEfSe-LDA analysis to identify significant biomarkers or features of the three experimental groups (Figure 14a,b). The LDA scores revealed that DSS treatment in mice led to an enrichment of 22 bacterial species, including *Clostridium* and *Escherichia*. On the other hand, SBP-FMT treatment in mice resulted in an enrichment of 40 bacterial profiles, including *Bacteroides* and *Bifidobacterium*, which was consistent with the effect of SBP treatment (Figure 14b). Moreover, through the examination of the primary microorganisms at the genus level in every sample and the observation of alterations in the primary microorganisms between the groups, we found that DSS treatment caused a notable reduction in *Prevotella* and *Allobaculum*, while simultaneously leading to an elevation in *Clostridium*, *Parabacteroides*, and *Escherichia*. However, SBP-FMT treatment was able to reverse these changes (Figure 14c,d). The results strongly indicate that the use of SBP-FMT therapy has a notable effect on the composition and variety of the intestinal microbiome, similar to the effects of SBP treatment. Therefore, we can infer that the modulation of SBP on the gut microbiota plays a decisive role in ameliorating colitis in mice.

### 3.12. Effects of SBP-FMT on SCFAS

**Figure 15:**
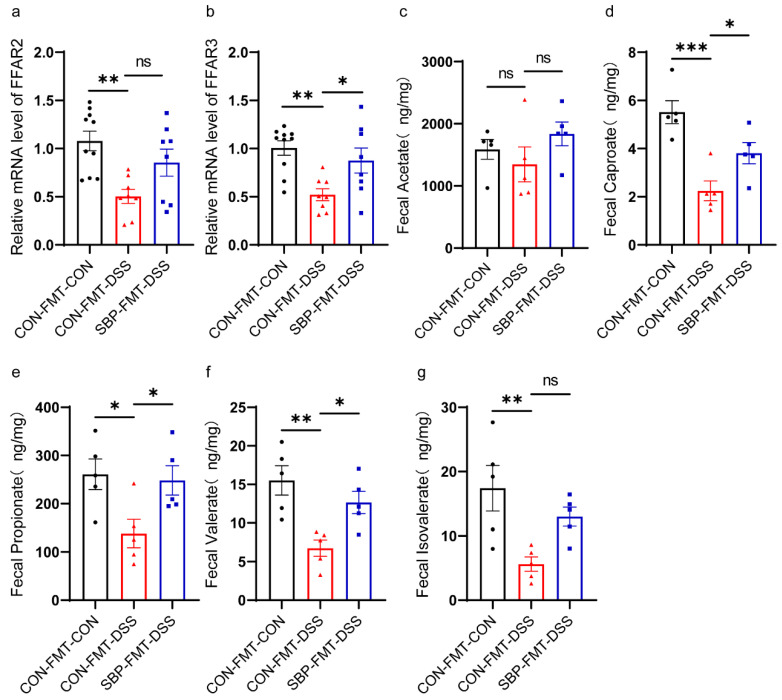
SBP-FMT improves the production of SCFAs. The relative expression of FFAR2 (**a**) and FFAR3 (**b**) in colon quantified by qPCR (n = 8–10). Concentrations of fecal acetate (**c**), caproate (**d**), isovalerate (**e**), propionate (**f**) and valerate (**g**) (n = 5). * *p* ≤ 0.05, ** *p* ≤ 0.01. *** *p* ≤ 0.001.

We investigated the impact of SBP-FMT on SCFAs levels. DSS treatment greatly decreased the mRNA levels of FFAR2 and FFAR3, but SBP-FMT partially reversed this effect (Figure 15a,b). The metabolomic analysis results indicated that acetate did not exhibit a noteworthy alteration with DSS treatment or SBP-FMT treatment (Figure 15c), whereas propionate, caproate, and valerate concentrations were decreased due to DSS treatment and restored by SBP-FMT treatment (Figure 15d–f). Isovalerate levels were also reduced by DSS treatment and restored by SBP-FMT, although not to a significant extent (Figure 15g). The findings suggest that SBP-FMT treatment also triggers the production of SCFAs.

## 4. Discussion

IBD is influenced by genetic predispositions and environmental triggers, with diet and gut microbiota being central factors. Dysbiotic alterations in the gut microbiota observed in IBD highlight the significance of the microbiota in disease pathogenesis and suggest the potential for targeting the microbiota as a therapeutic approach in IBD management [1,31,32]. The diet’s impact on microbial diversity suggests that promoting beneficial bacteria could ameliorate IBD-related dysbiosis. Certain dietary components can help beneficial gut microbiota like *Bifidobacterium* and *Lactobacillus* to thrive, which is important for a healthy gut and immune system [12,33].

SCFAs, metabolic byproducts of gut microbial fermentation of dietary fibers, have been shown to modulate the development and progression of IBD [34,35]. SCFAs exert comprehensive immunomodulatory effects, influencing both innate and adaptive immune responses [36]. For example, SCFAs promote the differentiation and function of regulatory T cells (Tregs), which play a critical role in maintaining immune tolerance and suppressing excessive inflammation. SCFAs also inhibit the production of pro-inflammatory cytokines [35,37,38]. Through these mechanisms, SCFAs help to maintain intestinal barrier integrity, reduce epithelial permeability, and dampen the inflammatory response in the gut [12,13].

FMT is a procedure that transfers healthy gut microbiota from a donor to a patient to help balance the patient’s gut microbiota. It has been successful in treating repeated infections from a specific harmful bacteria called Clostridium difficile [39,40,41]. Because changes in gut microbiota are linked to IBD, FMT is being considered as a hopeful new way to treat this condition.

Sea buckthorn polysaccharide (SBP), a substance from the sea buckthorn plant, is noted for its health benefits [42]. It boosts immune system cells like macrophages and natural killer cells and can regulate immune reactions by affecting cytokine production. Because of these immune-regulating and antioxidant qualities [43,44,45,46], SBP is being looked at as a possible treatment for immune system diseases like IBD. Studies have found that SBP can alleviate colonic inflammation, help maintain the intestinal barrier function, and modulate the composition of the gut microbiota disrupted by a high-fat diet [25]. However, further investigation is required to clarify the roles and mechanisms of SBP’s effects on IBD.

In our study, we administered SBP orally for 21 days followed by the induction of colitis for 7 days. Prophylactic SBP treatment effectively attenuated colitis symptoms, including reduced DAI scores, histological damage, spleen-to-body weight ratio, and improved feed quality and body weight.

In IBD, the digestive system suffers from ongoing inflammation due to complex interactions between immune cells and inflammatory chemicals [47,48,49]. Immune cells like macrophages and neutrophils release inflammatory substances which then attract more immune cells, making the inflammation worse [50,51,52]. An imbalance between inflammation-promoting and inflammation-reducing substances can make this damage even more severe [53,54]. Our study found that prophylactic SBP significantly lowered these inflammatory substances, including IL-6, IL-1β, COX-2, CCL2, and CCL5. SBP also reduced the activity of genes and pathways known to drive inflammation, such as TLR-4 and NLRP3, which are critical in IBD’s development [55,56,57,58]. COX-2 is an enzyme that helps create substances leading to inflammation. High amounts of COX-2 can make inflammation and damage in the gut worse for people with IBD [59,60]. Chemokines CCL2 and CCL5 have important functions in attracting and stimulating macrophages during inflammation and are implicated in the development of various inflammatory conditions [61,62,63]. Increased MPO activity and levels of MPO-derived oxidative products are associated with various inflammatory diseases [64,65]. SBP also has been shown to decrease the levels of MPO, thereby potentially mitigating the enzyme’s excessive activity linked to inflammation. Interestingly, we also observed that the anti-inflammatory cytokine IL-10 levels were elevated in response to DSS-induced colitis and subsequently diminished following administration of SBP. This elevation of IL-10 post-DSS treatment is suggestive of a compensatory anti-inflammatory mechanism, potentially serving to mitigate the escalation of the inflammatory milieu. The reduction in IL-10 levels post-SBP treatment could be indicative of an amelioration of the inflammatory state, thereby obviating the requirement for such a compensatory response. The result demonstrated the potential regulatory influence of SBP on cytokine profiles within the context of colitis and highlight the potential homeostatic reinstatement afforded by this intervention.

Additionally, SBP increased the production of antioxidant enzymes, including T-AOD, T-SOD, and CAT, which protect against oxidative stress and inflammation in the colon and plasma. However, our study does not definitively determine whether the enhanced antioxidant capacity is a cause or a consequence of reduced inflammation. It is possible that SBP may exert a dual modulatory effect, enhancing antioxidant defenses that in turn help to control inflammatory responses, or vice versa.

When the intestinal barrier is damaged, harmful substances and bacteria can get through, leading to an immune response [49]. Tight junction proteins like occludin, claudin-1, and ZO-1 are important for keeping this barrier functioning properly [66,67,68,69]. Mucins, such as Muc2 and Muc3, help maintain mucosal barrier integrity [70,71,72]. CDX2 is a key factor needed for the health of the intestinal epithelium [73,74]. Our study found that SBP helped to repair the damage to the gut barrier caused by DSS. This was shown by an increase in expression of tight junction proteins and mucins. Alcian blue staining of the colonic mucosa, as well as immunohistochemistry and immunofluorescence analysis of Muc2, supported these findings.

Given the significant impact of the gut microbiota on colitis, we examined the variations in gut microbiota among different treatment groups of mice using 16S rDNA gene sequencing. IBD has been closely linked to decreased microbial diversity and alterations in gut microbiota composition. Both PCoA analysis and UPGMA cluster analysis revealed notable differences in the community composition of the microbiota between the DSS and CON groups. The ASVs Venn diagram and Shannon index indicated a decrease in microbial diversity in the DSS group compared to the CON group. SBP treatment led to an improvement in diversity and a shift in community composition. The LEfSe analysis identified distinct dominant bacteria in different treatment groups. *Escherichia* and *Turicibacter* were among the dominant genera of the microbiota in the DSS group. *Escherichia* is a versatile bacterium that includes both beneficial and pathogenic strains. Certain serotypes of *Escherichia coli* can cause a range of gastrointestinal illnesses [75,76]. Pathogenic *E. coli* strains possess virulence factors that enable them to attach to and invade host cells, produce toxins, and evade the immune system [77,78]. *Turicibacter*, a genus of bacteria within the phylum Firmicutes, is still being investigated for its role and significance in the gut microbiota. Some species of *Turicibacter* have been implicated in disease conditions, including obesity, IBD, and colorectal cancer [79,80]. In contrast, prophylactic SBP treatment resulted in an enrichment of *Bifidobacterium* and *Bacteroides*. Reduced abundance of *Bifidobacterium* has been linked to gastrointestinal disorders, including IBD and irritable bowel syndrome (IBS) [81]. *Bifidobacterium*, a bacteria genus belonging to the phylum Actinobacteria, is known for its capability to ferment complex dietary fibers, produce vitamins, and exhibit immunomodulatory properties [82]. *Bacteroides* species are considered advantageous due to their ability to produce SCFAs and contribute to immune homeostasis and defense against pathogens [83,84]. These findings suggest that SBP treatment leads to an increase in beneficial bacteria and a reduction in harmful bacteria.

Our findings indicate that SBP has an impact on modulating the gut microbiota community, particularly specific microbiomes. Furthermore, the beneficial microbiome is facilitated by SCFAs. SCFAs play a crucial role in gut health and have diverse effects on the host’s physiology [85]. The study showed that SBP treatment increased the relative expression of SCFA receptors FFAR2 and FFAR3 [86,87]. Metabolomics results revealed that SBP treatment had a positive impact on the production of SCFAs in the gut, particularly caproate, propionate, valerate, and isovalerate. These SCFAs were reduced as a result of DSS treatment, indicating a disruption in their production. While the specific functions and effects of caproate, valerate, and isovalerate are still being investigated, evidence suggests their potential benefits, including anti-inflammatory properties, immune modulation, and antimicrobial activity [36,88].

Our subsequent objective was to examine the effects of intestinal bacteria and their byproducts, facilitated by prophylactic SBP, on colitis through FMT. We induced experimental colitis in mice and performed FMT using fecal samples from control mice or mice treated with SBP. Consistent with the effects of SBP treatment, SBP-FMT relieved colitis symptoms, suggesting that the modulation of gut microbiota by SBP plays a substantial role in alleviating colitis. SBP-FMT significantly reduced the inflammatory response, oxidative stress, and intestinal barrier damage caused by DSS, comparable to prophylactic SBP. These findings provide evidence that the reduced effects are linked to the intestinal microbiome and its byproducts, which are influenced by SBP intervention.

As anticipated, SBP-FMT led to a notable increase in SCFAs-producing microorganisms, such as *Bifidobacterium* and *Bacteroides*, thereby enhancing SCFAs synthesis, including caproate, propionate, valerate, and isovalerate. The results demonstrated that the beneficial effects of SBP-FMT were comparable to those of SBP treatment.

**Figure 16:**
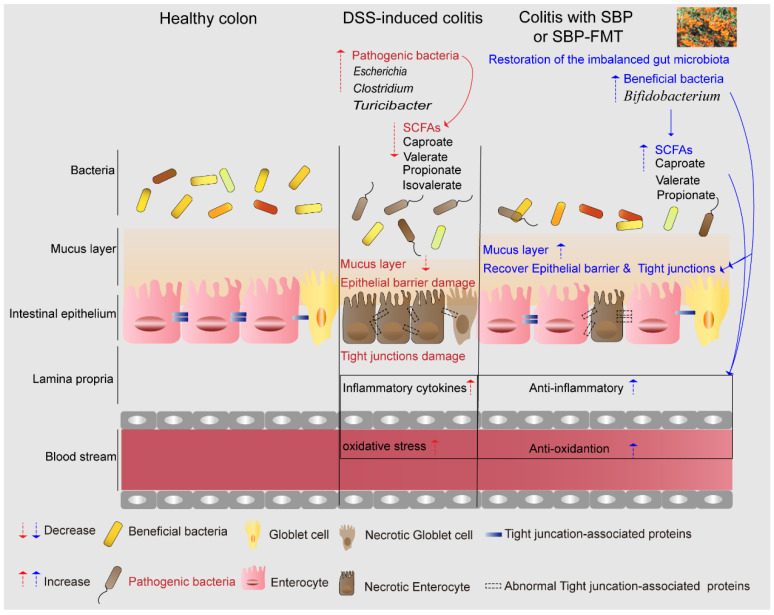
Mechanism of alleviation of DSS-induced colitis by prophylactic SBP or SBP-FMT. SBP exerts its effects on multiple aspects including the intestinal microbiota, oxidative stress, inflammation, and barrier integrity. Prophylactic SBP or SBP-FMT changed the composition of gut microbiota like enriching the probiotic bacteria Bifidobacterium and reducing the pathogenic bacteria Escherichia. This subsequently leads to an elevated production of SCFAs including valerate, isovalerate, and propionate. This increase in SCFAs triggered a cascade of beneficial effects, including anti-oxidative, anti-inflammatory, and barrier-protective responses. Ultimately, restoration of intestinal epithelial homeostasis and attenuation of colitis symptoms were achieved.

This study showcased that the modulation of the microbial community through SBP has a clear impact on alleviating colitis. Specifically, it led to the enrichment of beneficial bacteria, such as *Bifidobacterium* and *Bacteroides*, while reducing the abundance of harmful bacteria, such as *Escherichia*. The increased presence of SCFAs-producing bacteria and their metabolites, facilitated by SBP administration, contributed to the maintenance of colonic homeostasis. This metabolic shift promoted an antioxidative and anti-inflammatory state, providing protection against damage to the colonic barrier and mucosa. The summarized findings are presented in a pattern diagram (Figure 16). Overall, our results suggest that the induction of beneficial bacteria, particularly SCFAs-producing bacteria, and the reduction of pathogenic bacteria by SBP intervention contribute to an increase in short-chain fatty acids, thereby promoting an antioxidative and anti-inflammatory state and protecting against colonic barrier and mucosal damage.

## 5. Conclusions

Our study demonstrates the therapeutic potential of Sea Buckthorn Polysaccharide (SBP) in managing colitis by modulating gut microbiota and enhancing beneficial bacterial strains like *Bifidobacterium* and *Bacteroides*. This modulation leads to an increase in protective SCFAs, reducing inflammation and bolstering the intestinal barrier. The effects of SBP, corroborated by fecal microbiota transplantation experiments, suggest its role in promoting gut health and offering a non-pharmacological approach to IBD management. Our findings advocate for further clinical investigation of SBP as a dietary intervention for IBD, highlighting its potential to restore microbial balance and alleviate gastrointestinal inflammation.

## Figures and Tables

**Table 1:** Primer sequences used in RT-qPCR assays.

Target Gene	Forward Primer (5′-3′)	Reverse Primer (5′-3′)
CAT	CCTCGTTCAGGATGTGGTTT	TCTGGTGATATCGTGGGTGA
Ccl2	TACAAGAGGATCACCAGCAGC	ACCTTAGGGCAGATGCAGTT
Ccl5	TGCTGCTTTGCCTACCTCTC	TCTTCTCTGGGTTGGCACAC
CDX2	CTGGACAAGGACGTGAGCAT	ACTGCGGAGGACTGACAAAG
Claudin-1	CCTGCCCCAGTGGAAGATTT	CTTTGCGAAACGCAGGACAT
COX2	CCCATTAGCAGCCAGTTGTC	CAGGATGCAGTGCTGAGTTC
FFAR2	TACTGATCCGCAATCCTGCC	CACCCCTGTCCATCTTGGTC
FFAR3	CGACTAGAGATGGCTGTGGT	AGAAGATGAGCAGTGTGGCT
GAPDH	AGGTCGGTGTGAACGGATTTG	TGTAGACCATGTAGTTGAGGTCA
GSH-px	CCTCAAGTACGTCCGACCTG	CAATGTCGTTGCGGCACACC
IFN-γ	AGCAAGGCGAAAAAGGATGC	TCATTGAATGCTTGGCGCTG
IL-1β	AGCTTCAAATCTCGCAGCAG	TCTCCACAGCCACAATGAGT
IL-6	TAGTCCTTCCTACCCCAATTTCC	TTGGTCCTTAGCCACTCCTTC
IL10	TGAATTCCCTGGGTGAGAAGC	GACACCTTGGTCTTGGAGCTTA
Muc2	TCCTGACCAAGAGCGAACAC	ACAGCACGACAGTCTTCAGG
Muc3	GCTGGCTTTCATCCTCCACT	CCTCCATCCCACACACTTCC
NLRP3	TATCCACTGCCGAGAGGTGA	TCTTGCACACTGGTGGGTTT
Occludin	CCTCCACCCCCATCTGACTA	TCGCTTGCCATTCACTTTGC
SOD1	GGAACCATCCACTTCGAGCA	CTGCACTGGTACAGCCTTGT
TLR-4	CACCAGGAAGCTTGAATCCCT	GGAATGTCATCAGGGACTTTGC
TNF-α	TCCCAGGTTCTCTTCAAGGGA	GGTGAGGAGCACGTAGTCGG
ZO-1	GAGCCCCCTAGTGATGTGTG	TAGGGTCACAGTGTGGCAAG

## Data Availability

Raw data for qPCR, kits, and related statistics can be found here: https://figshare.com/s/817ef618d20441c9ff80.
